# Tumor response and survival in patients with advanced non-small-cell lung cancer: the predictive value of chemotherapy-induced changes in fibrinogen

**DOI:** 10.1186/1471-2407-12-330

**Published:** 2012-08-01

**Authors:** Jun Zhao, Mingfang Zhao, Bo Jin, Ping Yu, Xuejun Hu, Yuee Teng, Jingdong Zhang, Ying Luo, Lingyun Zhang, Shuang Zheng, Qiyin Zhou, Heming Li, Yunpeng Liu, Xiujuan Qu

**Affiliations:** 1 Department of Medical Oncology, The First Hospital, China Medical University, Shenyang, China

**Keywords:** Hyperfibrinogenemia, Biomarker, NSCLC

## Abstract

**Background:**

Hyperfibrinogenemia is a common problem associated with various carcinomas, and is accompanied by hypercoagulablity. In advanced non-small-cell lung cancer (NSCLC) it remains unclear whether or not chemotherapy-induced changes in fibrinogen level relate to chemotherapeutic response and prognosis. The purposes of this study were to: 1) analyze the association between chemotherapy-induced changes in plasma fibrinogen level and the chemotherapeutic response after the first two courses of standard first-line platinum-based chemotherapy; and 2) evaluate the prognostic significance of the basal plasma fibrinogen level in patients with advanced NSCLC.

**Methods:**

In this retrospective study, the data from 160 patients with advanced NSCLC were collected. The association between the changes in fibrinogen and the response to chemotherapy, or between the pre-and post-chemotherapy fibrinogen levels and patient clinical characteristics, were analyzed using SPSS software. In addition, the prognostic value of pre-chemotherapy fibrinogen levels was assessed.

**Results:**

The median pre-chemotherapy plasma fibrinogen level was 4.4 g/L. Pre-chemotherapy plasma fibrinogen levels correlated significantly with gender (p = 0.041). Post-chemotherapy plasma fibrinogen levels correlated with gender (p = 0.023), age (p = 0.018), ECOG (p = 0.002) and tumor response (p = 0.049). Plasma fibrinogen levels markedly decreased after chemotherapy in 98 (61.25 %) patients with pre-chemotherapy hyperfibrinogenemia (p = 0.008); and in this population there was a significant link between the decrease in fibrinogen level, and initial partial response (PR; p = 0.017) and stable disease (SD; p = 0.031). Univariate and multivariate analysis revealed that higher levels of fibrinogen (≥4.4 g/L) and ECOG 1 were positively associated with shorter overall survival (OS). CEA and CA125 also decreased significantly (p =0.015, p =0.000) in DCR group after chemotherapy.

**Conclusions:**

This study showed that the reduction in plasma fibrinogen levels induced by chemotherapy might be as a promising biomarker as CEA and CA125 for evaluating the efficacy of chemotherapy in advanced NSCLC. In addition, basal plasma fibrinogen levels could be used as an independent prognostic parameter for the OS of patients with advanced NSCLC.

## Background

Non-small cell lung cancer (NSCLC) is the leading cause of death from cancer worldwide. Platinum-based chemotherapy is currently the standard therapeutic strategy for advanced NSCLC [1]. However, the response rate is still about 24–42 %, suggesting that most NSCLC patients will not benefit from chemotherapy [1,2]. The patients who do not respond to chemotherapy will not only suffer from the additional adverse effects of chemotherapy, but also develop a progressively worse performance status. So it is very important to identify a chemotherapeutic predictor in order to avoid unnecessary treatment. To date, it has been reported that excision repair cross-complementary 1 (ERCC1) could predict the response to cisplatin [3,4], and that ribonucleotide reductase subunit M1 (RRM1) could predict the response to gemcitabine [5]. However, studies on these predictors are still ongoing, and conclusions as to their effectiveness have not yet been reached.

At present, serum tumor markers such as CEA, CYFRA21-1 and CA125 are commonly used markers for evaluating response to chemotherapy and prognosis in patients with malignancy. Not only serum markers but also hemostatic parameters have been examined as predictive factors of the efficacy of chemotherapy and prognosis. The specific relationship between coagulation parameters, such as D-dimer and platelets, and malignancy has been investigated for more than a century. Previous work has demonstrated that increased levels of D-dimer are indicative of disease progression and poor prognosis in lung, breast, esophageal and gastric colorectal cancers, as well as being a good predictor of survival and tumor progression [6-10]. On the other hand, thrombocytosis has been frequently observed in neoplastic diseases, especially in primary lung cancer, and pre-treatment platelet count has been shown to be an independent prognostic factor of survival [11]. Fibrinogen is also one of the important coagulation parameters, which is synthesized effectively in the liver and can be converted to fibrin by thrombin during the coagulation cascade [12]. Fibrinogen usually functions as an acute-phase protein with a normal concentration of 2 to 4 g/L in the plasma, increasing in level in response to most forms of wound healing, infection, inflammation or generation of tumor stroma [13-15]. It is increasingly recognized that basal fibrinogen levels can be used as an independent prognostic parameter in lung cancer, as well as pancreatic cancer, gastric cancer, colon cancer, esophageal cancer, gynecological malignancies and renal cancer [16-27]. Recent studies have also shown that a relationship might exist between plasma fibrinogen level and response to therapy in small cell carcinoma of the lung (SCCL) [28-30]. However, whether the basal plasma fibrinogen level, or changes in the level between the pre- and post-chemotherapy period can be used to evaluate the response to chemotherapy in patients with advanced NSCLC still remains unclear.

The aim of present study was to investigate the possible relationship between basal plasma fibrinogen levels and clinicopathological features, and the association between chemotherapy-induced changes in fibrinogen level and response to chemotherapy in patients with advanced NSCLC. The effects on progression-free survival (PFS) and overall survival (OS) were also analyzed. The results could provide a new approach with regard to the assessment of chemotherapeutic response and survival.

## Methods

### Patient selection

One hundred sixty patients, who were diagnosed with primary advanced NSCLC at the First Hospital of China Medical University (Liaoning, China) from February 2006 to November 2010, were enrolled in the present retrospective study. Patients were eligible for the study if they met the following criteria: 1) had been histologically or cytologically diagnosed as having NSCLC; 2) had non-resectable, recurrent or metastatic NSCLC; 3) had received no previous adjuvant chemotherapy, or were recurrent metastatic NSCLC patients who had not received adjuvant chemotherapy for at least 6 months; 4) had completed at least two cycles of chemotherapy; 5) had been tested for fibrinogen level before (pre-chemotherapy) and after two courses of chemotherapy (post-chemotherapy); 6) had an Eastern Cooperative Oncology Group performance status of 0 or 1; and 7) had adequate hepatic, renal, hematologic and cardiac function. Patients with severe acute or chronic inflammatory diseases, coagulation disturbances, chronic liver diseases, chronic renal failure or oral anticoagulation therapy were ruled out of this study. Approval for the study was obtained from the First Hospital of China Medical University Ethics Committee.

All of the patients were treated according to the standards applied to platinum-based chemotherapy. RESIST version 1.1 was used for the evaluation of tumor response. The final response to chemotherapy was classified into four categories: CR (complete remission); partial response (PR); stable disease (SD); and progression of disease (PD). CR, PR and SD were defined as the disease control rate (DCR). Each patient received follow-up visits.

### Follow-up

After all of the courses of chemotherapy had been completed, patients were followed up every 6 weeks until death. At closure of the study, dead or alive status was recorded for all patients. After a median follow-up time of 289 days (range, 51–1564 days), 106 patients had died by the cutoff date.

### Methods

Recurrent or metastatic disease was diagnosed pathologically, or by the presence of a measurable lesion on CT scan and bronchoscopy, or by CT-guided biopsy. Non-operative staging was performed according to the classification established by the American Joint Committee on Cancer (AJCC) version 7.

All patients with advanced NSCLC received platinum-based chemotherapy according to the severity of their respective conditions. Each patient received 2–6 courses of chemotherapy, and each course lasted for 3 weeks. An assessment was made after the first two courses of chemotherapy. Throughout chemotherapy, adjuvant medicine such as antanacathartic, colony stimulating factor and diphosphonate were given to reduce adverse events when necessary.

Coagulation and tumor marker related examination is one of the routine procedures carried out on cancer patients in our hospital. Pre-chemotherapy blood samples were obtained from patients within 1 week prior to chemotherapy, and post-chemotherapy samples were obtained after the first two courses of chemotherapy. The citrated blood samples were taken before breakfast and measured early in the morning. The STA Fibrinogen Kit is intended for the quantitative determination of the fibrinogen level in blood plasma using the Clauss clotting method. The reference range of plasma fibrinogen level was defined as being between 2 and 4 g/L using this method. The manufacturer claims an intra-assay CV is 2.3 %-3.7 %. Hyperfibrinogenemia was defined as a plasma fibrinogen concentration of >4 g/L. Serum levels of CEA and CA125 were considered normal at levels between 0–4.3 ng/ml and 0–35 U/ml, respectively.

All the records were reviewed and the following data were collected: gender; stage; histological type; Eastern Cooperative Oncology Group (ECOG) score; age; response to chemotherapy after the first two courses; ORR (objective response rate), PFS; OS; and fibrinogen levels. PFS was calculated from the date of diagnosis to disease progression (local or metastatic) or death. OS was calculated from the date of diagnosis to the date of death or the last follow-up.

### Statistical analysis

All statistical analyses in this study were carried out using the SPSS 17.0 statistical package for Windows. The continuous data was expressed as the mean ± standard deviation and the categorical data was expressed as percentages (%). The relationship between plasma fibrinogen and clinicopathological factors, or plasma fibrinogen and response to chemotherapy, was evaluated using the Mann–Whitney U test, Kruskal–Wallis test or the Spearman rank correlation coefficient where appropriate. The χ^2^ test was used for qualitative data analysis. The Kaplan–Meier curve was used to describe PFS and OS, and differences between groups were compared by means of the log-rank test. Univariate analysis comprised sex (male vs. female), age (<60 years vs. ≥60 years), histological type (adenocarcinoma vs. squamous cell carcinoma vs. other tumor types), ECOG score (0 vs. 1), cancer stage (III vs. IV) and fibrinogen levels (<4.4 g/L vs. ≥4.4 g/L). All statistical analysis parameters presenting significant correlation or a tendency towards association (P <0.20) with the outcomes of interest were entered into multivariable logistic regression analysis. Results were analyzed for the end points of PFS and OS. A P value <0.05 was considered as being statistically significant in all statistical analyses.

## Results

### Patient characteristics

The mean value of pre-chemotherapy plasma fibrinogen level in the 160 patients studied was 4.72 ± 1.46 g/L. The general characteristics of the study population are summarized in Table 1. Of the 160 patients who received chemotherapy, 102 (63.8 %) were male and 58 (36.2 %) were female. Ages ranged between 28 and 77 years, with a median age of 57 years. Pre-chemotherapy fibrinogen level was found to be significantly correlated with gender (p = 0.041), and post-chemotherapy fibrinogen level was correlated with age (p = 0.018), gender (p = 0.023) and ECOG score (p = 0.002).

**Table 1:** Association between pre- and post-chemotherapy fibrinogen levels and clinical characteristics

**Characteristics**		**pre-chemotherapy fibrinogen**		**pre-chemotherapy fibrinogen**	
	**N (%)**	**(g/L) (mean ± SD)**	**p value**	**(g/L) (mean ± SD)**	**p value**
**Age**					
<60	95(59.4 %)	4.58 ± 1.28	0.053	4.44 ± 1.27	0.018
≥60	65(40.6 %)	5.06 ± 1.65		4.79 ± 1.27	
**gender**					
Male	102(63.8 %)	4.91 ± 1.53	0.041	4.72 ± 1.21	0.023
Female	58(36.2 %)	4.40 ± 1.29		4.34 ± 1.25	
**Stage**					
III	60(37.5 %)	4.895 ± 1.68	0.497	4.61 ± 1.35	0.851
IV	100(62.5 %)	4.639 ± 1.32		4.57 ± 1.24	
**Histological type**					
Adenocarcinoma	98(61.2 %)	4.69 ± 1.41	0.358	4.60 ± 1.28	0.677
Squamous carcinoma	34(21.3 %)	5.06 ± 1.72		4.36 ± 1.16	
Others	28(17.5 %)	4.41 ± 1.28		4.78 ± 1.39	
**ECOG**					
0	105(65.6 %)	4.55 ± 1.38	0.051	4.33 ± 1.11	0.002
1	55(34.4 %)	5.04 ± 1.57		5.07 ± 1.44	

Spearman rank correlation coefficient analysis also revealed a significant correlation between the fibrinogen level, white blood cell (WBC) count, platelet count and hemoglobin level. Higher plasma fibrinogen was often observed to be significantly correlated with increased WBC and platelet counts, and reduced hemoglobin level.

### Fibrinogen levels and chemotherapy response

**Figure 1:**
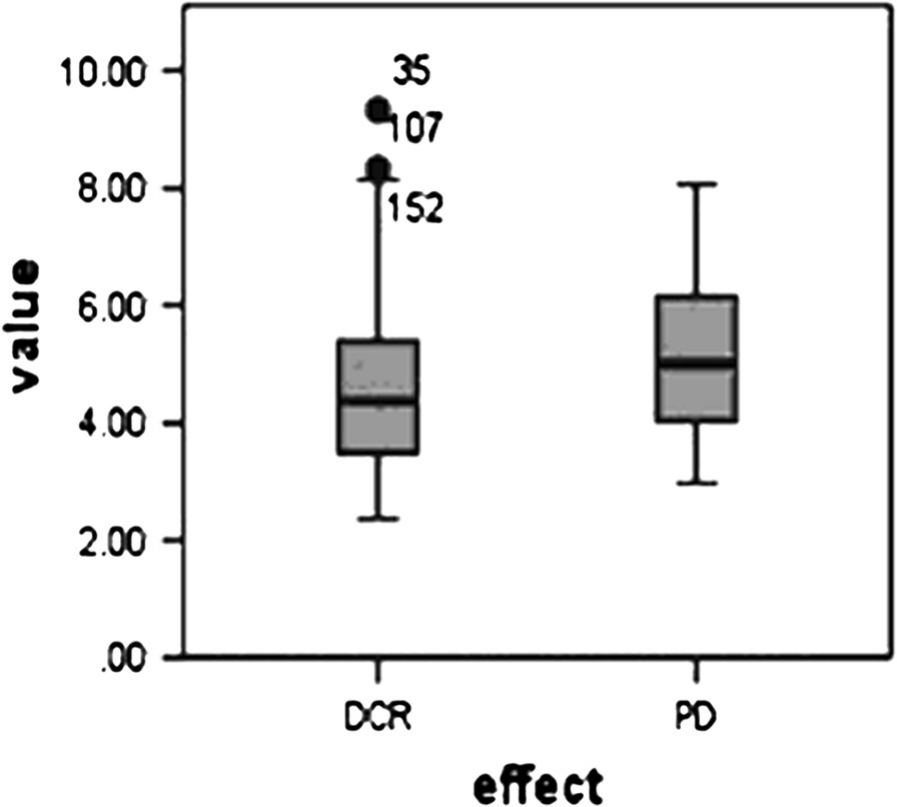
**Comparison of pre-chemotherapy fibrinogen levels in terms of the disease control rate (DCR) and progression of disease (PD).** p = 0.128.

**Figure 2:**
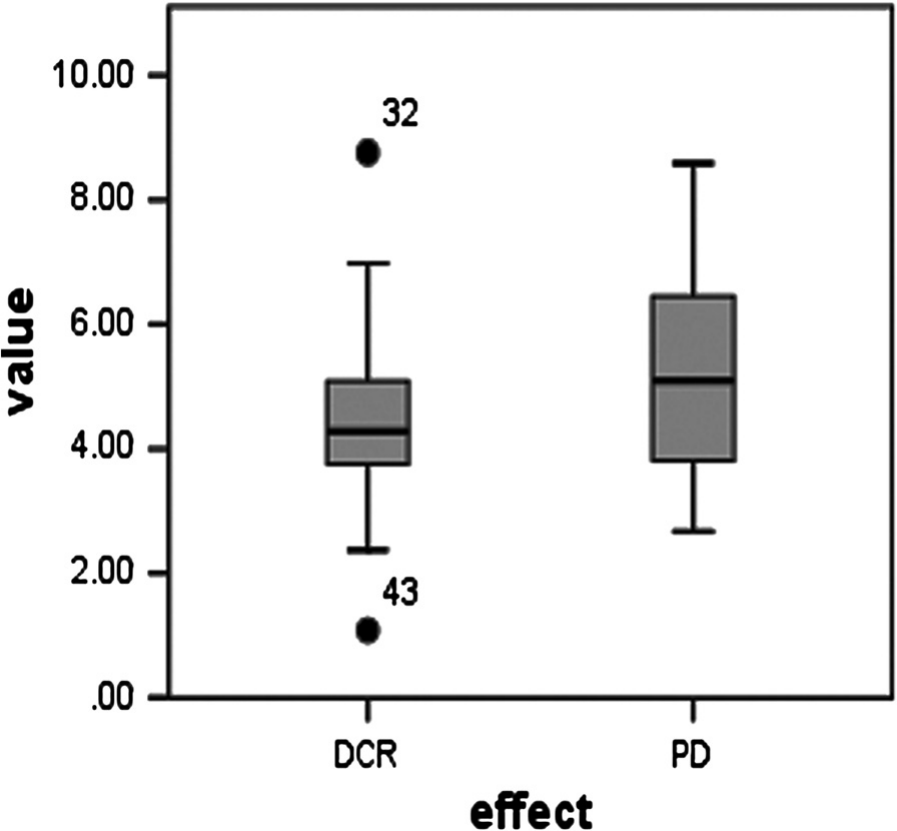
**Comparison of post-chemotherapy fibrinogen levels in terms of the disease control rate (DCR) and progression of disease (PD).** p = 0.049.

The relationship between pre-chemotherapy fibrinogen levels and treatment response rates is detailed in Table 2; in 160 patients the pre-chemotherapy fibrinogen level had no significant effect on the PR, SD or PD (χ^2^ = 3.188; p = 0.203). Clinical benefit, namely DCR defined as a CR, PR or SD, was recorded in 81.88 % of patients in this study (PR, 22.5 %; SD, 59.4 %). The CR, PR, SD and PD were 0(0 %), 36(22.5 %), 95(59.4 %), 29(18.1 %), respectively. The mean pre-chemotherapy fibrinogen levels were 4.64 ± 1.44 g/L and 5.09 ± 1.52 g/L for DCR and PD patient groups, respectively, but they did not reach statistical significance (Figure 1; p = 0.128). Interestingly, post-chemotherapy fibrinogen levels differed significantly between the DCR (4.46 ± 1.15 g/L) and PD (5.15 ± 1.65 g/L) groups (Figure 2; p = 0.049).

**Table 2:** Effect of pre-chemotherapy fibrinogen level on chemotherapy response

	**Fibrinogen high**	**Fibrinogen low**	**Total**
PR	21(13.1 %)	15(9.4 %)	36(22.5 %)
SD	55(34.4 %)	40(25 %)	95(59.4 %)
PD	22(13.8 %)	7(4.3 %)	29(18.1 %)

Low Fibrinogen ≤4 g/L.

High fibrinogen >4 g/L.

P = 0.203.

**Figure 3:**
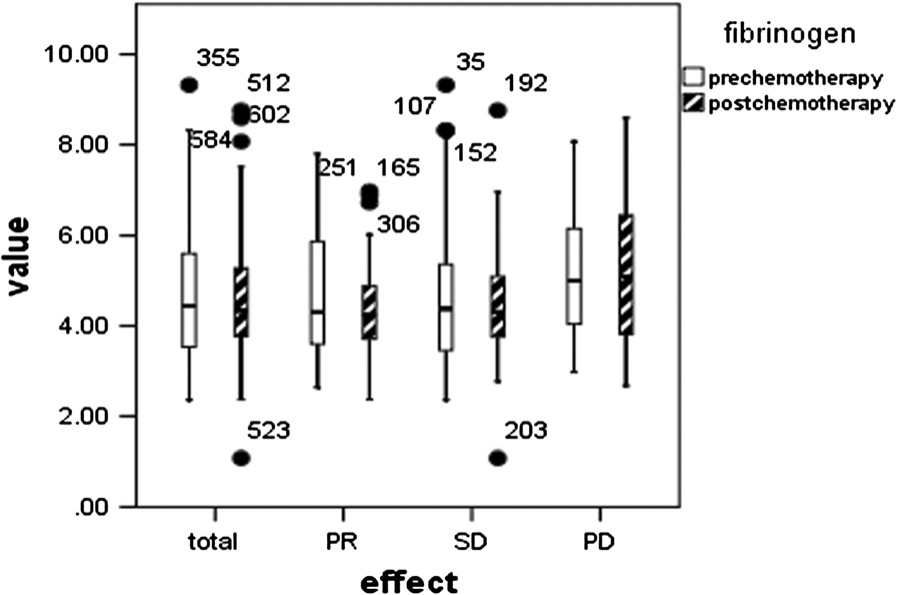
**Comparison between pre- and post-chemotherapy fibrinogen levels in 160 patients.** Total: p = 0.805, PR: p = 0.432, SD: p = 0.935, PD: p = 0.802.

The comparison between pre-and post-chemotherapy fibrinogen levels in 160 patients is shown in Figure 3. The pre-chemotherapy plasma fibrinogen level in the 160 patients was 4.72 ± 1.46 g/L, and although it had decreased to 4.58 ± 1.27 g/L after chemotherapy, the decline in level was not significant (p = 0.805). Similarly, there was no association between pre-chemotherapy fibrinogen levels and chemotherapy response.

**Figure 4:**
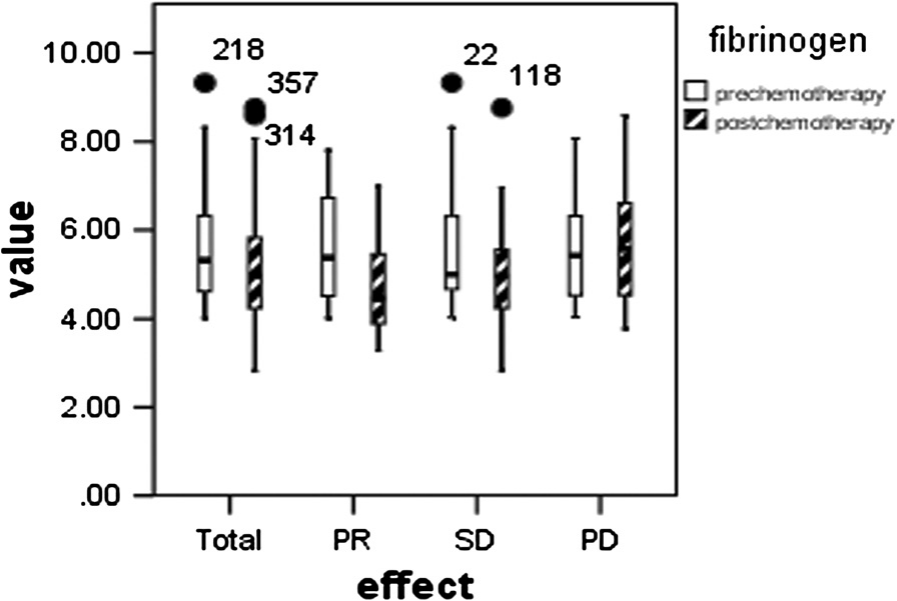
**Comparison between pre- and post-chemotherapy fibrinogen levels in 98 patients with pre-chemotherapy hyperfibrinogenemia.** Total: p = 0.008, PR: p = 0.017, SD: p = 0.031, PD: p = 0.689.

We also investigated whether a difference in hyperfibrinogenemia existed in patients pre- and post-chemotherapy. As shown in Figure 4, pre-chemotherapy hyperfibrinogenemia was found in 98 (61.3 %) patients with a mean level of 5.58 ± 1.22 g/L, and it decreased to 5.12 ± 1.22 g/L after chemotherapy (p = 0.008). We then evaluated changes in pre- and post-chemotherapy fibrinogen levels in these 98 patients in terms of PR, SD and PD. Pre-chemotherapy fibrinogen levels were 5.68 ± 1.23, 5.51 ± 1.22 and 5.68 ± 1.26 g/L, and post-chemotherapy fibrinogen levels were 4.82 ± 1.15, 4.97 ± 1.11 and 5.76 ± 1.39 g/L in the PR, SD and PD groups, respectively. Post-chemotherapy fibrinogen levels were significantly lower than pre-chemotherapy levels in the PR (p = 0.017) and SD (p = 0.031) patient groups, but were not higher in patients in the PD group (p = 0.689). Thus, the change in fibrinogen levels between pre-chemotherapy and post-chemotherapy might be more useful in evaluating chemotherapy response in the patients with pre-chemotherapy hyperfibrinogenemia.

### Fibrinogen levels and prognosis

**Figure 5:**
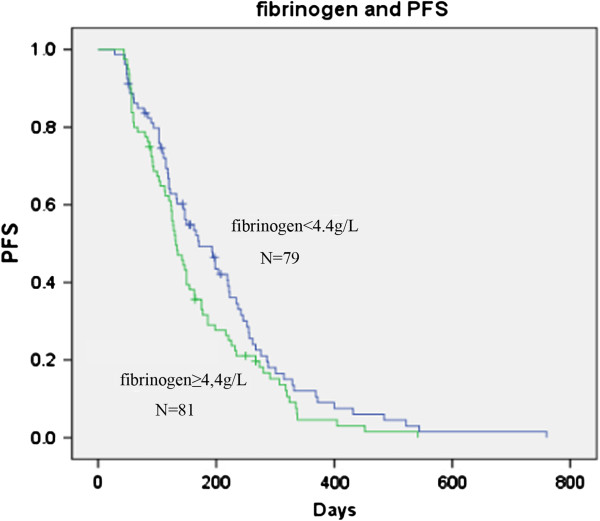
**Kaplan–Meier curves for progression-free survival (PFS) broken down by median plasma fibrinogen level (4.4 g/L) in 160 patients.** p = 0.085.

**Figure 6:**
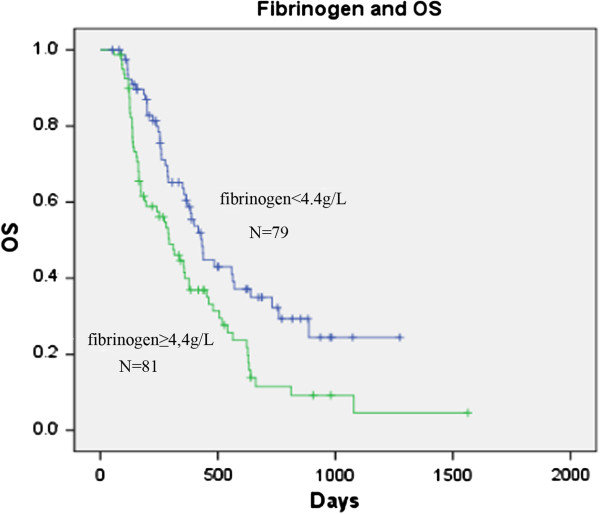
**Kaplan–Meier curves for overall survival (OS) broken down by median plasma fibrinogen level (4.4 g/L) in 160 patients.** p = 0.003.

In order to evaluate the value of fibrinogen as a prognostic factor, the relationship between fibrinogen, and PFS and OS was analyzed. According to the method used by Ferrigno et al. and Buccheri et al., patients were divided into two groups, one above and the other below the median pre-chemotherapy plasma fibrinogen level of 4.4 g/L [16,17]. The data showed that although patients with hyperfibrinogen had a shorter PFS time than those without hyperfibrinogen (132 days vs. 171 days), there was no significant difference between the two groups (Figure 5; p = 0.085). Interestingly, the occurrence of a higher fibrinogen level showed a significant correlation with shorter survival time. The median survival time was 292 days for patients in the high pre-chemotherapy fibrinogen group versus 432 days for patients in the low pre-chemotherapy fibrinogen group (Figure 6; p = 0.003). Furthermore, we evaluated the prognostic value of the change in fibrinogen levels between pre-and post-chemotherapy. Ninety eight pre-chemotherapy hyperfibrinogenemia patients were divided into two groups, according to the change in pre-and post-chemotherapy fibrinogen levels (increased level group and decreased level group after chemotherapy; cut-off value Δf = 0 g/L). PFS and OS were not significantly different between the increased fibrinogen group and the decreased fibrinogen group (data not shown).

Multivariate analysis indicated that ECOG and pre-chemotherapy fibrinogen levels were independent predictors of OS. ECOG 1 was found to be an inferior predictor of PFS relative to fibrinogen (Table 3). Cox regression analysis showed that other factors like age, sex, stage of disease and histological type were not associated with OS and PFS (P >0.05).

**Table 3:** Multivariate analysis

**Variable**	**Adverse covariate**	**Relative risk**	**95 % CI**	**P**
ECOG	1	1.691	1.110-2.576	0.014
Fibrinogen	≥4.4 g/L	1.673	1.133-2.471	0.01

95 % CI indicates 95 % confidence interval.

ECOG = Eastern Cooperative Oncology Group score.

### CEA and CA125 levels and chemotherapy response

As shown in Table 4, pre-and post-chemotherapy CEA levels were measured in 145 patients and 88 of these patients (60.69 %) presented with elevated levels. Post-chemotherapy CEA levels were significantly lower (p = 0.015) than pre-chemotherapy levels in the DCR group, but were comparable (p = 0.35) in the PD group (Table 4). As shown in Table 5, pre-and post-chemotherapy CA125 levels were measured in 114 patients and 69 patients (55.2 %) presented with elevated levels. Post-chemotherapy CA125 levels were significantly lower (p = 0.000) than pre-chemotherapy levels in the DCR group, but were comparable (p = 0.322) in the PD group (Table 5).

**Table 4:** Alterations in CEA levels induced by chemotherapy in 145 NSCLC patients

**Group**	**N (%)**	**pre-chemotherapy (ng/ml)**	**post-chemotherapy (ng/ml)**	**P value**
**Total**	145	96.74 ± 242.48	76.14 ± 208.93	0.082
**DCR**	116	100.8 ± 253.19	74.25 ± 211.46	0.015
**PD**	29	80.51 ± 196.80	83.68 ± 201.92	0.35

**Table 5:** Alterations in CA125 levels induced by chemotherapy in 114 NSCLC patients

**Group**	**N (%)**	**pre-chemotherapy (U/ml)**	**post-chemotherapy (U/ml)**	**P value**
**Total**	114	120.00 ± 186.15	99.05 ± 194.99	0.002
**DCR**	93	104.63 ± 169.92	67.75 ± 113.21	0.000
**PD**	21	188.10 ± 238.74	237.68 ± 362.12	0.322

### CEA and CA125 levels and prognosis

In 149 patients with baseline CEA levels, the PFS was 150 days for groups with both normal and elevated levels of CEA (p = 0.545). The OS was 416 and 356 days in patients with normal and high levels of tumor marker, respectively (p = 0.89) (Data not shown).

**Figure 7:**
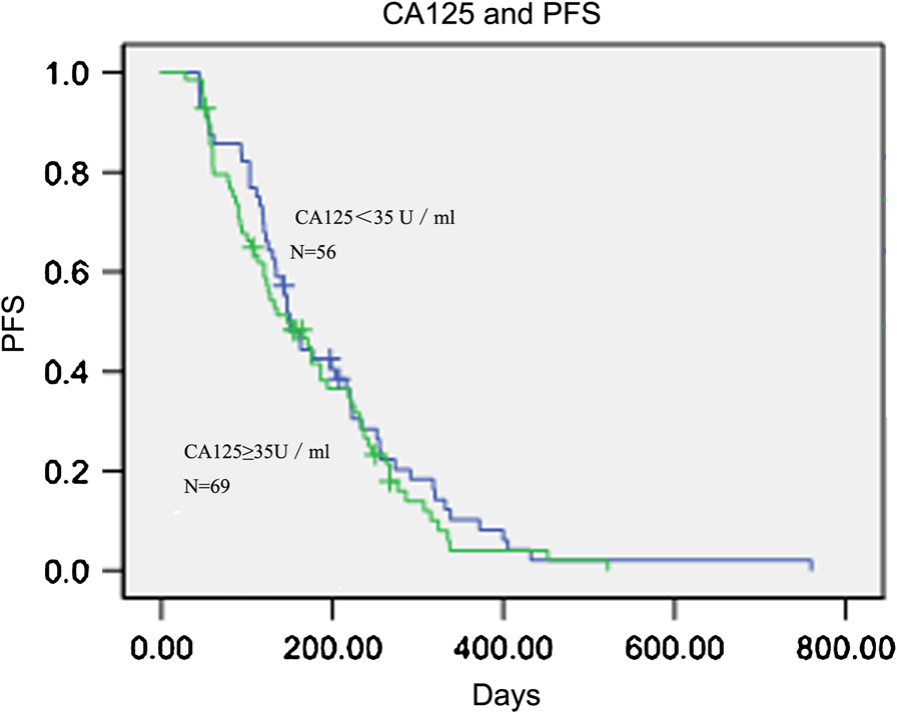
**Kaplan–Meier curves for progression-free survival (PFS) broken down by CA125 upper normal limit (35 U/ml) in 125 patients.** p = 0.431.

**Figure 8:**
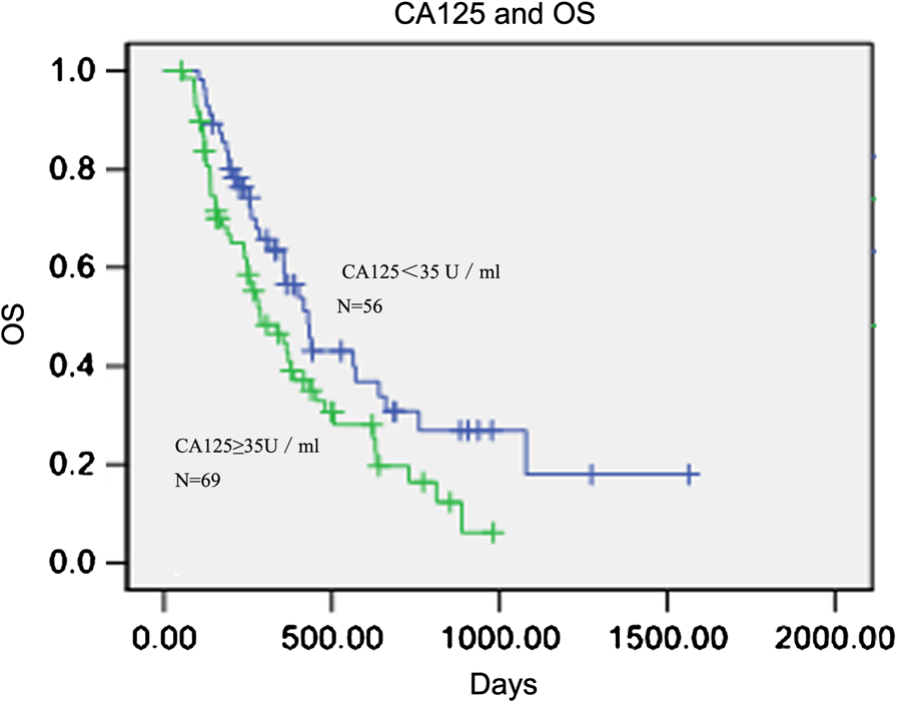
**Kaplan–Meier curves for progression-free survival (PFS) broken down by CA125 upper normal limit (35 U/ml) in 125 patients.** p = 0.029.

In 125 patients with baseline CA125 levels, the PFS was 150 and 147 days in groups with normal and elevated levels of CA125, respectively (p = 0.431) (Figures 7). The OS was 429 and 288 days in patients with normal and high levels of tumor marker, respectively (p = 0.029) (Figures 8). However, only CA125 levels were found to be significant predictive factors for OS when evaluated using multivariate analysis (p = 0.006).

## Discussion

Fibrinogen is one of the important parameters concerning the relationship between malignancy and coagulation disorders. A link between hyperfibrinogenemia and malignant diseases has previously been revealed. It is increasingly recognized that fibrinogen levels have prognostic significance in several cancers. In our study, we assessed the effect of plasma fibrinogen levels on advanced NSCLC in 160 patients.

To our knowledge, previous studies related to fibrinogen in tumors were mainly focused on its prognostic value [16-27], while there have been few studies where the fibrinogen level has been used for evaluating response to chemotherapy [23,28,29]. There are two studies in which the relationship between fibrinogen level and therapeutic response have been assessed in SCCL. The first study was carried out on 37 patients, and revealed that patients not responding to chemotherapy had higher fibrinopeptide A levels than those who responded to chemotherapy. In contrast, patients with a prolonged CR did not exhibit evidence of abnormal thrombin activity [28]. In the second study carried out on 119 patients, higher pre-treatment fibrinogen levels at diagnosis reflected a more advanced stage of disease, and were also associated with a reduced likelihood of a positive response to chemotherapy and a lower OS. This effect was more apparent in SCCL patients with limited stage disease [29]. In NSCLC, a small study including 49 patients with unresectable locally advanced or metastatic lung cancer has been carried out. The data showed that the average basal levels of fibrinogen were high but remained constant after chemotherapy in these 49 NSCLC patients [30]. Our study analyzed the relationship between the basal level of fibrinogen and chemotherapy-induced changes in fibrinogen, and chemotherapy response. The results showed that fibrinogen levels decreased significantly in 98 patients with pre-chemotherapy hyperfibrinogenemia, but the decrease was not significant in the 160 patients. This was, in part, in accordance with findings for other malignancies. The potential reasons for this where that the pre-chemotherapy fibrinogen level in some of the patients that responded to chemotherapy was normal, and may not have significantly decreased after chemotherapy. In addition, the sample size was small. Therefore, these findings should encourage the initiation of further large scale trials to confirm the hypothesis that elevated fibrinogen can represent poor responsiveness to chemotherapy in advanced NSCLC.

In previous large multicenter studies involving patients with epithelial ovarian cancer and endometrial cancer, it was reported that pre-therapeutic plasma fibrinogen levels could be used as an independent prognostic parameter for disease-free and overall survival [24,25]. Tang et al. found that high pre-operative plasma fibrinogen levels were associated with distant metastases and impaired prognosis, after curative resection in patients with colorectal cancer [22]. Yamashita et al. suggested that the pre-operative plasma fibrinogen level could be a useful predictor of lymphatic metastasis in intestinal-type gastric cancer [20]. A study involving 93 patients with NSCLC who underwent surgical resection demonstrated that high plasma fibrinogen levels were correlated with increasing tumor size, advanced pathological T stage, squamous cell carcinoma and a poor prognosis. Another study that enrolled patients with advanced NSCLC demonstrated that reduced survival was associated with higher fibrinogen levels [18]. In our study, similar results raise the possibility that the pre-chemotherapy plasma fibrinogen level was associated with OS in NSCLC. This is in accordance with previously published data in patients with other malignancies, reflecting the fact that fibrinogen can be used as an independent prognostic parameter for survival.

The proliferative characteristics of tumor cells and the interaction with different stromal cells and supportive tissue govern how the tumors grow. It has been determined that most solid tumors contain a substantial amount of fibrinogen and fibrin, the fibrous protein into which fibrinogen is transformed. This is frequently deposited around solid tumors, suggesting that fibrinogen may promote the formation of tumor stroma. Along with other adhesive glycoproteins, fibrinogen forms a coating on the surfaces of tumor cells, and serves as a scaffold to support the binding of growth factors and to promote tumor cell dispersion in a manner analogous to wound repair. Simultaneously, the coating of fibrinogen could also protect tumor cells from immune surveillance and prolong malignant cell survival [31].

It is a general opinion that advanced cancer and anorexia-cachexia syndrome are often associated with an inflammatory response [32,33]. Increasing age is linked to a higher state of subclinical inflammation and an increased production of proinflammatory cytokines [26]. Patients enrolled in the present study were all diagnosed with advanced NSCLC. Elderly patients and those with weaker performance status seem to have higher levels of plasma fibrinogen. Fibrinogen level was significantly correlated with WBC and platelet counts in our study. This is probably evidence that high fibrinogen levels are indicative of, not only a coagulation factor but also an acute-phase reactant protein that is greatly enhanced in response to infection and other inflammatory disorders. However, the precise mechanisms involved have not been fully clarified. Interleukin-6 (IL-6), which is believed to be the key cytokine, is capable of stimulating hepatocyte fibrinogen synthesis [34]. In contrast, another important inflammatory cytokine, IL-1, has been shown to inhibit fibrinogen synthesis [35]. Higher fibrinogen levels have often been observed to be significantly associated with reduced hemoglobin levels, indicating that hypoxia might induce an increase in fibrinogen levels. Thus, the regulation of fibrinogen synthesis during inflammation appears to be quite complicated.

The role of the serum tumor markers in advanced NSCLC is controversial [36,37]. Several reports have been published concerning the prognostic value of CEA; there have been fewer reports on CA-125[38,39]. CEA is a serum tumor marker that is expressed in numerous solid tumors. There are discordant findings with regard to the accuracy of pretreatment levels of CEA in predicting prognosis in advanced NSCLC patients. In a study by Jin et al. involving 111 advanced NSCLC patients, CEA level was found to be useful in evaluating chemotherapy response, and baseline CEA level was shown to be a significant predictive factor for OS [40]. Susana et al. reported on 277 advanced NSCLC patients where high baseline levels of CEA were found to be a significant negative prognostic factor [38]. Similar results have been reported by other authors [41,42]. However, the results of other studies have indicated that CEA level was not useful in the prognostic evaluation of NSCLC patients [43]. Similarly, in the studies of Kulpa et al. and Ardizzoni et al. involving 107 patients with advanced-stage squamous cell lung cancer and 200 patients with stage I-IV non small cell lung cancer, respectively, CEA was not found to be a prognostic factor for survival [36,37]. In our current study, the change in CEA levels before and after chemotherapy was correlated with therapeutic response (DCR group) and we did not find that it was a significant prognostic factor for OS.

The role of CA125 in NSCLC is not well known. Trapé et al. reported that CA125 could be a predictive factor for response to treatment and a prognostic factor for survival in patients with NSCLC treated with chemotherapy [39]. In a study by Susana et al., CA125 level was found to have increased in approximately half of the population and this marker had a significant prognostic value [38]. In our study, CA125 levels decreased significantly after chemotherapy in the DCR group and had prognostic value for OS.

## Conclusions

First, higher pre- and post-chemotherapy plasma fibrinogen levels were associated with male gender, but were not associated with unfavorable histological type. Furthermore, elevated post-chemotherapy fibrinogen levels were also significantly associated with ECOG 1 and increased patient age. Second, chemotherapy significantly reduced fibrinogen levels in patients with pre-chemotherapy hyperfibrinogenemia. The reduction also showed a good correlation with tumor regression induced by chemotherapy in DCR patients. However, it did not relate to longer PFS or OS. Third, the elevated pre-chemotherapy plasma fibrinogen levels were significantly associated with shorter OS in 160 patients, with a trend towards a similar association for PFS. Finally, CEA and CA125 levels decreased significantly after chemotherapy in the DCR group. CA125 level at baseline can be used as an independent prognostic parameter in advanced NSCLC patients. The results presented in the present study support the idea that fibrinogen could be as a useful biomarker as CEA and CA125 for evaluating the response of advanced NSCLC to chemotherapy. Pre-chemotherapy fibrinogen can also be used as an independent prognostic parameter in patients with advanced NSCLC. These findings highlight the potential benefit of a new therapeutic strategy. This will entail the future development of practical methods for the inhibition of fibrinogen that will be evaluated in large scale studies.

## Competing interests

The authors declare that they have no competing interests.

## Authors’ contributions

JZ analyzed the data and wrote the manuscript. MZ, BJ, PY, XH, YT, JZ, YL, LZ and HL collected the data of the patients. SZ and QZ followed up the patients. YL and XQ designed the research, edited the manuscript, and supported the project. All authors read and approved the final manuscript.

## Authors’ information

All of the authors belong to Department of Medical Oncology, The First Hospital, China Medical University

## Pre-publication history

The pre-publication history for this paper can be accessed here:

http://www.biomedcentral.com/1471-2407/12/330/prepub
